# Psychiatrist and Peritoneal Dialysis

**DOI:** 10.1192/j.eurpsy.2022.1916

**Published:** 2022-09-01

**Authors:** D. Klarić, V. Klarić, M. Klarić

**Affiliations:** 1 Ob Zadar, Nephrology And Dialysis, Zadar, Croatia; 2 Faculty of Medicine, Rijeka, Medicine, Zadar, Croatia

**Keywords:** peritoneal, dialysis, therapy, group

## Abstract

**Introduction:**

Peritoneal dialysis (PD) is an equal method of treating patients with end stage renal disease ( ESRD). The patients are left to themselves in the new situation. The psychiatrist recognizes their needs and through group therapy enables them to heal quality intrapsychic conflicts.

**Objectives:**

The study analized data on the intensity of depression, anxiety of an individual patient respectively, but also of his family member (caregiver) too. The control questionnaires are foreseen for both groups one year after group therapy participation. The assumption is that symptoms of depression and anxiety will be less expressed with group support by the psychiatrist.

**Methods:**

Two questionnaires were used: Hamilton’s rating scale for depression and Hamilton’s anxiety rating scale and identical questionnaires for member of the family caring for the patients. 13 patients who accepted group therapy were examined in our institution. They were of different gender and age, mean age 53±13.46 mini-max 25-72 years.

**Results:**

Average months of dialysis duration 29.15±21.84 min-max 6-84 Dialysis was performed without an assistant but with some help n 13( 100%) from the patient. They describe ailments from anxiety (30.77%) and depression ( 38.46% ) which they did not have premorbidly, and feel rejected on the emotional sphere, although not on the part of organic medicine.

**Conclusions:**

Emphasis is placed on the emotional state and needs of the patient with severe physical ailments, in other words, demanding treatment methods, as well as the importance of emotional support from family members without whom these patients would have a poorer quality of life.

**Disclosure:**

No significant relationships.

